# Surpassing kilometer-scale terahertz wireless communication beyond 300 GHz enabled by hybrid photonic–electronic synergy

**DOI:** 10.1038/s41377-026-02390-7

**Published:** 2026-06-26

**Authors:** Mikhail Gorbun, Georgy Fedorov

**Affiliations:** Department of Physics and Mathematics, Center for Photonics Sciences, University of Eastern Finland, Joensuu, Finland

**Keywords:** Microwave photonics, Optoelectronic devices and components, Terahertz optics

## Abstract

An important milestone in the path towards the 6 G wireless communication systems is presented in a recent work demonstrating a record-breaking THz wireless communication at a net rate of 27.84 Gbit/s over a 2.2 km wireless link using a carrier frequency of 335 THz lying in the most challenging atmospheric window.

Next-generation wireless data transfer systems are doomed to operate at carrier frequencies beyond 100 GHz, i.e., in the sub-terahertz frequency range^1,2^. Despite the evident fact that increasing carrier frequency allows for higher data transfer rates there are several challenges hampering sub-THz data transfer networks. The major challenge is related to atmospheric radiation absorption. Atmosphere attenuates radiation in the sub-terahertz frequency range, with water vapor being the primary absorber. As a result, data transmission at these frequencies is highly challenging and is only possible within so-called transmission windows^3^, bands lying between strong absorption lines. Even within these windows the absorption remains significant. This settles the requirement for emission power.

At present, the output power of terahertz-wave generation is typically limited to hundreds of microwatts, which is insufficient to compensate for atmospheric losses. Solid-state amplifiers are used to increase the emitted power. Their main drawback is that their saturation output power is generally limited to the milliwatt range^4,5^.

The recent research by Yuancheng Cai and co-authors addressed^6^ some of these challenge in their study published in *Light: Science & Applications* and demonstrated record breaking data transfer line operating at 50GBt/sec through a distance of more than 2 km. This line uses 335 GHz carrier frequency. This carrier frequency allows for data transfer at the rate far beyond 100GBt/sec involving polarization-division multiplexing and quadrature amplitude modulation technologies.

There are several break-throughs facilitating this result. First of all, the research group significantly improved the radiation source they had designed and reported in their previous works. The key element of the innovative source is a self-developed continuous-wave traveling wave tube amplifier (TWTA) module. This 66 TWTA module is designed with a transformative folded waveguide (FWG) slow-wave structure (SWS), which enables a continuous output power up to 3.82 W and a signal gain of over 50 dB^7,8^. The photonics-assisted THz generation approach based on O/T conversion is used to generate high-frequency and high-rate THz signals, and the high-gain continuous-wave TWTA is used to amplify the THz signal power enough to counteract the link losses of long-range THz wireless transmission. The advanced geometry of the TWTA module allows for an unprecedented power output. Optical to THz conversion principle enables high modulation rate (See the Fig. 1).

**Fig. 1:**
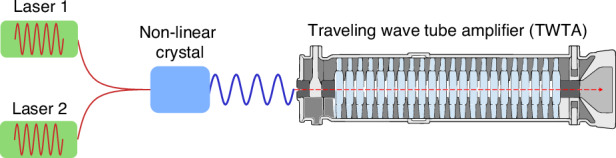
Schematics of the THz source

The combination of photonic generation and electronic amplification is particularly effective: the high-gain continuous-wave TWTA boosts the THz signal to power levels sufficient to compensate for the substantial propagation and atmospheric losses that typically limit long-range THz wireless communication. At the same time, the use of optical-to-THz conversion allows for flexible and high-speed modulation, making it well suited for next-generation ultra-high-capacity wireless systems. Together, these innovations create a powerful and efficient platform for long-distance THz transmission (See the Fig. 2).

**Fig. 2:**
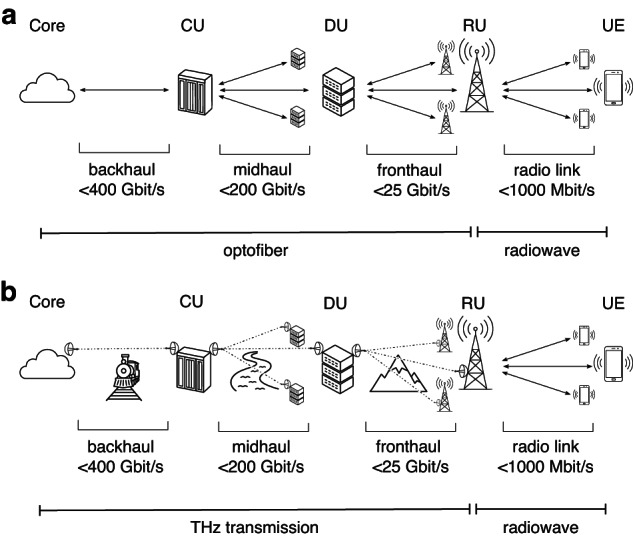
Concept of 5 G and 6 G networks. **a** Relying mostly on optical fibers. **b** Using the terahertz wireless communication

Sensitivity on the detection side is enhanced by using a diversity reception approach in which a single transmitter sends the signal to two separate receivers. Specifically, two electronics-based THz mixing receivers are placed 5 cm apart behind a PTFE lens to improve detection performance. Each receiver includes a horn antenna, a THz low-noise amplifier, and an integrated harmonic mixer, and both units are powered by the same RF source.

Starting with 5 G networks, the data flow from the user equipment (UE) to the core network typically passes through a radio unit (RU), that is, the antenna system at the base station. From the RU, the traffic is forwarded to a distributed unit (DU), which connects several RU, coordinates their operation, and supports functions such as handover. The data are then transmitted to a central unit (CU), which provides coordinated control over multiple DU. The CU is, in turn, connected to the core network^9,10^.

Nowadays, in the majority of transmission segments between RU, DU, CU and core links are physically realized using optical fiber. These are the segments that are considered to be replaced with THz wireless data transmission. It could enable connectivity in locations where the deployment of optical fiber is impeded by urban infrastructure, natural obstacles, or legally protected architectural heritage sites and ecosystems.

The signal-to-noise ratio and the bandwidth achieved in this work allows for significant increase of the data transfer far beyond 100Gbit/sec^11,12^.
